# Cost analysis of pharmaceutical care provided to HIV-infected patients: an ambispective controlled study

**DOI:** 10.1186/s40199-014-0074-5

**Published:** 2015-02-10

**Authors:** Renata Cavalcanti Carnevale, Caroline de Godoi Rezende Costa Molino, Marília Berlofa Visacri, Priscila Gava Mazzola, Patricia Moriel

**Affiliations:** Department of Clinical Pathology, Faculty of Medical Sciences (FCM), University of Campinas (UNICAMP), Alexander Fleming, 105, 13083-881 Campinas, SP Brazil; Faculty of Pharmaceutical Sciences (FCF), University of Campinas (UNICAMP), Sérgio Buarque de Holanda, 25, 13083-859 Campinas, SP Brazil

**Keywords:** Pharmacoeconomics, Pharmaceutical care, HIV-infected patients

## Abstract

**Background:**

Studies have shown that pharmaceutical care can result in favorable clinical outcomes in human immunodeficiency virus (HIV)-infected patients, however, few studies have assessed the economic impact. The objective of this study was to evaluate the clinical and economic impact of pharmaceutical care of HIV-infected patients.

**Methods:**

A controlled ambispective study was conducted in Brazil from January 2009 to June 2012. Patients were allocated to either intervention or control group. The control group was followed according to standard care while the intervention group was also followed by a pharmacist at each physician appointment for one year. Effectiveness outcomes included CD4+ count, viral load, absence of co-infections and optimal immune response, and economic outcomes included expenses of physician and pharmaceutical appointments, laboratory tests, procedures, and hospitalizations, at six months and one year.

**Results:**

Intervention and control groups included 51 patients each. We observed significant decreases in total pharmacotherapy problems during the study. At six months, the intervention group contained higher percentages of patients without co-infections and of patients with CD4+ >500 cells/mm^3^. None of the differences between intervention and control group considering clinical outcomes and costs were statistically significant. However, at one year, the intervention group showed higher percentage of better clinical outcomes and generated lower spending (not to procedures). An additional health care system daily investment of US$1.45, 1.09, 2.13, 4.35, 1.09, and 0.87 would be required for each additional outcome of viral load <50 copies/ml, absence of co-infection, CD4+ >200, 350, and 500 cells/mm^3^, and optimal immune response, respectively.

**Conclusion:**

This work demonstrated that pharmaceutical care of HIV-infected patients, for a one-year period, was able to decrease the number of pharmacotherapy problems. However, the clinical outcomes and the costs did not have statistical difference but showed higher percentage of better clinical outcomes and lower costs for some items.

## Introduction

Since 1996, Brazil has had a public health system program that provides free antiretroviral therapy, laboratory tests, and procedures to HIV-infected patients. This program has been internationally recognized as a major initiative against HIV [1]. However, this program alone does not guarantee safety and effectiveness of treatment because HIV treatment requires long-term therapy. Treatment of HIV includes a large number of drugs and drug interactions, and requires careful monitoring of therapy, with the goal of decreasing viral resistance and drug-related problems [1-3].

Studies have investigated the effects of pharmaceutical care on the rational use of drugs in HIV-infected patients [4-6]. March et al. demonstrated that HIV-infected patients followed by a clinical pharmacist show significant improvements in CD4+ levels and viral load and a decrease in toxic effects related to treatment [7]. This reduction in toxicity improves the quality of life and treatment adherence of the patients [8]. In addition, a systematic analysis (including data from January 1980 to June 2011) revealed that providing pharmaceutical care to HIV-infected patients was associated with statistically significant improvements in treatment adherence and had a positive impact on viral suppression [9].

Despite the variety of pharmaceutical care studies conducted with HIV-infected patients, remarkably few include an economic analysis. There is a need to go beyond the investigation of clinical outcomes generated by pharmaceutical care. Studies that include the economic impact of pharmaceutical care are necessary to justify the implementation or expansion of pharmaceutical care services [10].

In addition to the lack of economic studies on pharmaceutical care conducted with HIV-infected patients, another limitation is that, even though the studies available demonstrate that pharmaceutical care practice can contribute to the reductions of costs, they focus only on the costs associated with drugs, physician appointments, and hospitalizations. The available literature does not present studies regarding the impact of pharmaceutical care on the costs associated with laboratory tests and procedures [9,11-13]. Moreover, the majority of pharmacoeconomic studies present many methodological limitations, such as the lack of a control group and non-inclusion of costs associated with pharmacist appointments [14]. Thus, it has become imperative to conduct well-designed studies in the area of pharmaceutical care in order to obtain a clearer comprehension of its economic impact [14]. Well-designed studies investigating economic impact should be encouraged because they enable the rationalization of resources in health care, where the available resources are limited [15].

This study was designed to perform a pharmacoeconomic analysis of the impact of pharmaceutical care on HIV-infected patients over a one-year period by measuring both clinical and health care system economic outcomes.

## Methods

This was a one-year, ambispective, controlled study, with a systematic sample by quota controls, paired according to random characteristics. A retrospective chart review and a prospective pharmaceutical care follow-up were conducted. The study was conducted at a hospital in the state of São Paulo, Brazil. The Hospital Ethics Committee approved the research, and informed consent was obtained from all patients.

The inclusion criteria for the study were as follows: outpatients diagnosed with HIV/AIDS (Human Immunodeficiency Virus/Acquired Immunodeficiency Syndrome), aged between 18 and 60 years, having body mass index (BMI) lower than 30 kg/m^2^, and receiving antiretroviral therapy (ART). Obese patients were not included because they present higher incidences of hyperlipidemia, hypertension, and insulin resistance, and because some HIV/AIDS medications such as protease inhibitors can cause weight gain and fat accumulation, it would not be possible to determine whether the weight gain was related to the medication or to the background disease in such patients [16-19]. Patients who were unable to return for later appointments/exams, who refused to participate, who have psychiatric disease (that unable them to follow the medical appointments schedule and the pharmacist interventions), and those who were pregnant were excluded. Patients were enrolled in the study from January 2009 to June 2011 and were assigned in a 1:1 ratio to either intervention or control group by the clinical pharmacy team. Control group patients were matched to intervention group patients according to gender and baseline CD4+ count.

For one year, the intervention group was followed by the clinical pharmacy staff, composed of two pharmacists trained by the hospital clinical pharmacy team regarding HIV/AIDS and pharmaceutical care, after routine medical appointments at the hospital, using a method developed and adapted to the reality of the hospital, based on the Pharmacist’s Workup of Drug Therapy (PWDT) method [20]. The control group was not followed by the clinical pharmacy team, and its data were collected through review of medical charts encompassing the same period.

Initial and final pharmacotherapy problems were accounted for and classified as necessity, effectiveness, safety, or therapy compliance pharmacotherapy problems only for the intervention group [21]. The clinical pharmacy team performed written and verbal pharmacist interventions with the intervention group, which were accounted for and classified as pharmacist-patient or pharmacist-physician interventions and as resolutive pharmacotherapy problems, preventive pharmacotherapy problems, quality of life, or referral to other medical specialties interventions. The classifications used for pharmacotherapy problems and pharmacist interventions are in accordance with those used in another publication by the authors [22].

The five effectiveness outcomes were as follows: CD4+ count higher than 200 cells/mm^3^, 350 cells/mm^3^, and 500 cells/mm^3^; viral load lower than 50 copies/ml; and absence of co-infections. The co-infections considered by the study were as follows: bacterial co-infections (urinary infection, shigellosis, infected sebaceous cysts, cellulitis, pneumonia, and hordeolums), viral co-infections (cytomegalovirus, influenza, *Herpes zoster*, *Herpes simplex*, human papillomavirus, viral conjunctivitis, and warts), parasitic co-infection (microsporidiosis, isosporiasis, coccidiosis, and neurocryptococcosis), and fungal co-infections (oral moniliasis, onychomycosis, and tinea pedis). Effectiveness outcomes were measured at six months and one year of study and were obtained through medical chart review for both groups. Additionally, using a decision tree model, we established the number of patients from both groups that achieved, after one year of study, an optimal immune response as characterized by viral load <50 copies/ml, absence of co-infection, and CD4+>500 cells/mm^3^.

For cost analysis, we identified the number of appointments (medical/nursing/nutrition/physical therapy/speech therapy/dental), laboratory tests, procedures, and hospitalizations per patient in the first six months, the last six months, and in one year, for both groups, through review of their medical charts. For the intervention group, we also included the cost of the pharmacist appointment. The DATASUS database [23] provided the monetary values for all these items. Values were quoted in US dollars ($).

Cost analysis was performed for the one-year period, considering both the effectiveness and the costs of appointments, laboratory tests, procedures, hospitalizations, total cost, and total cost without procedures.

Statistical analysis of the results was performed by SAS System for Windows (Statistical Analysis System, version 9.2). For baseline characteristics analysis, chi-square, Fisher’s exact, and Mann–Whitney tests were performed. For co-infection, CD4+ and viral load analysis and generalized estimating tests were performed. For costs analysis, ANOVA for repeated measures, with a transformation by positions, was performed. The significance level was set at 5% (P ≤0.050).

## Results

**Figure 1:**
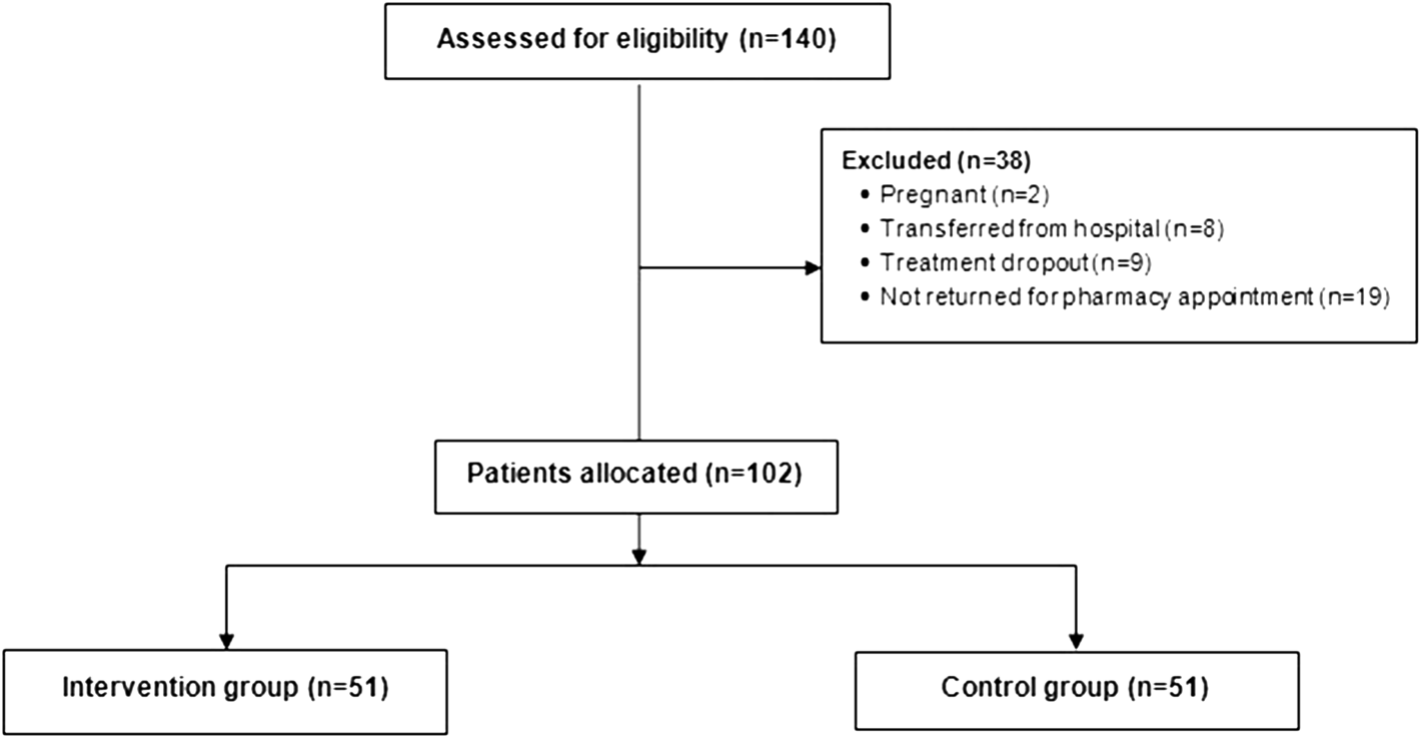
**Flow diagram of the patients included in the study.**

The study screened 140 HIV-infected patients being treated at the hospital on an outpatient basis. Thirty-eight patients were excluded: two were pregnant; nine interrupted the treatment at the hospital; eight were transferred from the hospital; and nineteen had not returned for the second pharmaceutical appointment in the first six months of the study (Figure 1). Finally, 51 patients each were allocated to intervention and control groups. A medical chart review provided the demographic data and initial information for both study groups (Table 1). The two study groups had similar baseline characteristics, indicating homogeneity.

**Table 1:** **Baseline characteristics of intervention group and control group**

**Characteristics**	**Control group**	**Intervention group**	***P*** **value**
	**N=51**	**N=51**	
**Age (Mean [SD], years)**	40.5 [9.2]	41.3 [8.8]	0.580^a^
**Men - % (n)**	66.7 (34)	66.7 (34)	1.000^b^
**Ethnicity - % (n)**			0.830^b^
Caucasian	70.6 (36)	68.6 (35)	
Black/ african descent	29.4 (15)	31.4 (16)	
**HIV Diagnosis** **(Mean [SD], years)**	7.5 [5.6]	8.3 [6.4]	0.690^a^
**HIV Treatment Duration (Mean [SD], years)**	5.7 [4.2]	6.5 [5.5]	0.780^a^
**Number of tablets/day (Mean [SD])**	9.3 [4.4]	10.1 [4.2]	0.250^a^
**ART changes during the first 4 weeks of the study - % (n)**	9.8 (5)	7.8 (4)	1.000^c^
**CD4 + (Mean [SD], cells/mm** ^**3**^ **)**	304.0 [277.1]	310.4 [302.0]	0.980^a^
CD4 + >200 cells/mm^3^ % (n)	56.9 (29)	54.9 (28)	0.840^b^
CD4 + >350 cells/mm^3^ % (n)	31.4 (16)	33.3 (17)	0.830^b^
CD4 + >500 cells/mm^3^ % (n)	15.7 (8)	17.7 (9)	0.800^b^
**Viral load <50 copies/ml % (n)**	60.8 (31)	64.8 (33)	0.190^b^
**Number of Comorbidities** ** (Mean [SD])**	2.5 [1.6]	2.8 [2.1]	0.730^a^
**Type of comorbidities % (n)**			
Hepatitis C	23.5 (12)	21.6 (11)	0.630^b^
Tobaccoism	15.8 (8)	11.8 (6)	0.570^b^
Neurotoxoplasmosis	9.8 (5)	9.8 (5)	0.510^b^
Hypertriglyceridemia	3.9 (2)	9.8 (5)	0.440^c^
Pulmonary tuberculosis	13.7 (7)	3.9 (2)	0.160^c^
**ART regimen % (n)**			0.800^b^
TDF+3TC+EFV	17.6 (9)	21.6 (11)	
AZT+3TC+EFV	15.7 (8)	21.6 (11)	
AZT+3TC+LPV/r	9.8 (5)	7.8 (4)	
TDF+3TC+LPV/r	11.8 (6)	11.8 (6)	
Others	45.1 (23)	37.2 (19)	
**Substance abuse % (n)**			0.418^b^
Alcohol	19.6 (10)	29.4 (15)	
Tobacco	27.4 (14)	33.3 (17)	
Illicit drugs	13.73 (7)	7.8 (4)	

Note: ^a^Mann-Whitney test; ^b^Chi-square test;^c^Fisher’s exact test.

*Abbreviations*: ART, antiretroviral therapy; AZT, zidovudine; CD4+, lymphocyte T CD4+; EFV, efavirenz; LPV/r, lopinavir/ritonavir; n, absolute number of patients; SD, standard deviation; TDF, tenofovir; 3TC, lamivudine.

There were a total of 230 pharmaceutical appointments (143 in the initial six months and 87 in the final six months). During these appointments, 219 pharmacist interventions were performed. Among them, 185 (84.5%) were pharmacist-patient interventions and 34 (15.5%) were pharmacist-physician interventions; 116 (53.0%) were preventive interventions, 55 (25.1%) were resolutive interventions, 42 (19.2%) were quality of life interventions, and six (2.7%) were referral to other medical specialties interventions. We also observed significant decreases in total pharmacotherapy problems (from 248 to 145; 41.5%, P <0.001), necessity problems (from 55 to 26; 52.7%, P <0.001), and safety problems (from 161 to 96; 40.4%, P <0.001). A decrease in the other pharmacotherapy problems was also detected; however, it was not statistically significant: effectiveness problems (from 12 to 11; 8.4%, P =1.0000) and compliance problems (from 20 to 12; 40.0%, P =0.760).

**Figure 2:**
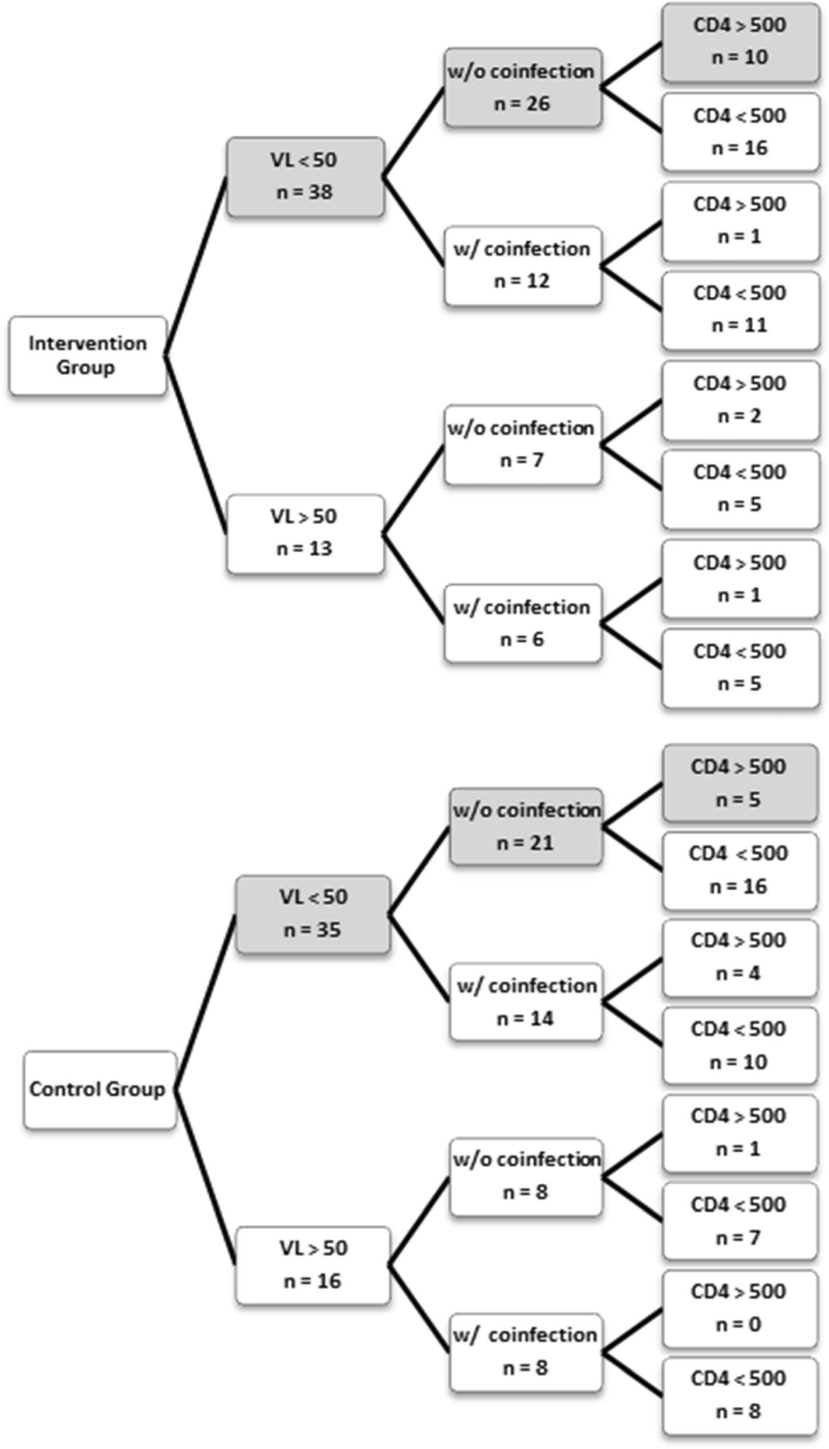
**Optimal response immune for control and intervention groups.** Abbreviation: CD4, CD4+ lymphocites; VL, viral load; w/o, without; w/, with.

Regarding clinical outcomes, in the initial six months, the intervention group contained higher percentages of patients without co-infections and of patients with CD4+ >500 cells/mm^3^. At one year, the intervention group showed higher percentage of better clinical outcomes: absence of co-infection, viral load <50 copies/ml, CD4+ >200 cells/mm^3^, CD4+ >350 cells/mm^3^, and CD4+ >500 cells/mm^3^ (Table 2). However, none of these differences was statistically significant. In addition, by using the decision tree model to establish the number of patients from each study group that achieved an optimal immune response (Figure 2), it was possible to infer that pharmaceutical care improves a patient’s immune response.

**Table 2:** **Co-infection, viral load and CD4+ at baseline, 6 months, and at one year of study**

	**Control Group**			**Intervention Group**			***P*** **value** ^**a**^
	**N=51**			**N=51**			
	**Basal**	**6 months**	**1 year**	**Basal**	**6 months**	**1 year**	
	**% (n)**	**% (n)**	**% (n)**	**% (n)**	**% (n)**	**% (n)**	
**Absence of co-infection**	/	72.6 (37)	56.9 (29)	/	76.5 (39)	64.7 (33)	0.092
**Viral load <50 copies/ml**	60.8 (31)	76.5 (39)	68.6 (35)	64.8 (33)	58.8 (30)	74.5 (38)	0.869
**CD4+>200 cells/mm** ^**3**^	56.9 (29)	68.6 (35)	74.5 (38)	54.9 (28)	70.8 (34)	78.4 (40)	0.793
**CD4+>350 cells/mm** ^**3**^	31.4 (16)	37.3 (19)	49.0 (25)	33.3 (17)	37.5 (18)	51.0 (26)	0.977
**CD4+>500 cells/mm** ^**3**^	15.7 (8)	17.7 (9)	19.6 (10)	17.7 (9)	20.8 (10)	27.5 (14)	0.599

Note: ^a^Statistical significance value - Generalized estimating equations (GEE) test.

*Abbreviation*: CD4+, lymphocyte T CD4+.

At six months, the intervention group presented with two bacterial, five viral, and two fungal co-infections and, at one year, presented with two bacterial, one viral, two parasitic, and one fungal co-infections. At six months, the control group presented with five bacterial, three viral, two parasitic, and one fungal co-infections, and at one year, presented with one bacterial, five viral, and three parasitic co-infections.

At one year of study, the intervention group spent less per day on appointments, laboratory tests, and hospitalizations, but spent more on procedures and in total than the control group. Moreover, only the intervention group spent on pharmaceutical appointments. Compared with the control group, the intervention group annually generated savings per patient of $3.20 associated with appointments, $23.19 with laboratory tests, and $5.94 with hospitalizations. The intervention group also generated additional annual costs per patient of $50.60 associated with procedures, $12.88 with pharmaceutical appointments, and $31.13 with total costs (Table 3). However, the difference in costs between the groups was not statistically significant. The stark contrast in the costs associated with procedures was caused by two hip surgeries performed on patients from the intervention group, which together added $1,916.09 to the total expenses. This amount corresponds to 48.0% of the total spent on procedures in the first six months of the study ($3,991.04). Excluding the costs of these procedures from the total costs, the results demonstrate that compared with the control group, the intervention group would have spent $19.40 less per patient per year (Table 3).

**Table 3:** **Study length of time and total daily costs (US$) for control and intervention groups**

	**Control Group**			**Intervention Group**		
	**N=51**			**N=51**		
**Study period**	**6 m (i)**	**6 m (f)**	**Final**	**6 m (i)**	**6 m (f)**	**Final**
Length of time (mean [SD], days)	180.2[41.8]	190.5[6.4]	370.7[41.3]	196.8[29.3]	190.2[56.4]	387.0[39.8]^a^
**Appointments**						
Total cost (US$) /day	8.65	6.49	**7.53**	8.01	6.12	**7.08**
Individual mean [SD] (US$)	0.17[0.08]	0.13[0.09]	**0.15[0.07]**	0.16[0.09]	0.12[0.08]	**0.14[0.07]**
**Laboratory tests**						
Total cost (US$) /day	38.07	25.35	**31.48**	33.33	22.96	**28.24**
Individual mean [SD] (US$)	0.75[0.74]	0.50[0.46]	**0.62[0.43]**	0.65[0.61]	0.45[0.43]	**0.55[0.44]**
**Procedures**						
Total cost (US$) /day	3.75	4.81	**4.29**	20.28	2.11	**11.36**
Individual mean [SD] (US$)	0.07[0.22]	0.09[0.21]	**0.08[0.17]**	0.40[1.11]	0.04[0.09]	**0.22[0.57]**
**Hospitalization**						
Total cost (US$) /day	2.62	2.53	**2.57**	2.30	1.17	**1.74**
Individual mean [SD] (US$)	0.05[0.30]	0.05[0.18]	**0.05[0.17]**	0.05[0.13]	0.02[0.10]	**0.03[0.09]**
**Pharmacist**						
Total cost (US$) /day	0.0	0.0	**0.0**	2.20	1.39	**1.80**
Individual mean [SD] (US$)	NA	NA	**NA**	0.05[0.02]	0.03[0.02]	**0.04[0.02]**
**Total**						
Final cost/day (US$)	53.09	39.18	**45.88**	66.13	33.74	**50.23**
Individual mean [SD] (US$)	1.04[0.99]	0.77[0.70]	**0.90[0.60]**	1.30[1.33]	0.66[0.55]	**1.00[0.81]**
**Total excluding procedures**						
Final cost/day (US$)	49.34	34.36	**41.58**	45.84	31.63	**38.87**
Individual mean [SD] (US$)	0.97[0.94]	0.67[0.63]	**0.82[0.56]**	0.90[0.71]	0.62[0.05]	**0.77[0.53]**

Note: ^a^ ANOVA results for repeated measures with a transformation by positions (P=0.057).

*Abbreviations*: 6m (i), initial 6 months; 6m (f), final 6 months; SD, Standard deviation.

Obs. The cost is the sum of the daily costs for all patients.

Cost analysis identified the additional costs associated with procedures and total costs required to achieve each of the clinical outcomes outlined in the study (Table 4). Moreover, we found that, for each $1.00 spent on pharmaceutical care, there was a loss of $1.42 per day. However, when the costs associated with procedures were excluded from final costs, for each $1.00 spent on pharmaceutical care, there was a benefit of $2.51 per day. No relationship could be identified between the total daily costs generated by the patients and the reductions of pharmacotherapy problems (P =0.292; correlation R =0.15039; Spearman correlation coefficient test), or between the total costs and the number of pharmacist interventions (P =0.706; correlation R = −0.05412; Spearman correlation coefficient test).

**Table 4:** **Incremental Cost Effectiveness Ratio analysis per day for procedures and total costs (US$)**

**For each additional outcome of:**	**ICER (US$/day)**	**ICER (US$/day)**
	**Procedures**	**Total Cost (with procedures)**
Viral load <50 copies/ml	2.36	1.45
Absence of co-infection	1.77	1.09
CD4+>200 cells/mm^3^	3.53	2.18
CD4+>350 cells/mm^3^	7.07	4.35
CD4+>500 cells/mm^3^	1.77	1.09
Optimal immune response	1.41	0.87

*Abbreviation*: ICER, Incremental Cost Effectiveness Ratio.

## Discussion

Pharmaceutical interventions were mostly of the pharmacist-patient type, which prevented therapy compliance errors (i.e., the patients needed clarifications regarding the use of medication, especially regarding dosage, drug interactions, and adherence). This type of intervention can help increase patient adherence to therapy. Hirsch et al. demonstrated in a cohort study with 2,234 patients that patients undergoing pharmaceutical care had greater adherence to antiretroviral therapy than patients not undergoing pharmaceutical care [6].

The decreases observed for all pharmacotherapy problem types are consistent with the literature. Studies have shown that pharmacist interventions can effectively identify, prevent, and solve pharmacotherapeutic problems [24,25]. Problems relating to safety were the most frequently encountered in our study. Other researchers have also identified a high frequency of issues related to inappropriate dosage and safety [5,26-29]. Carcelero et al. demonstrated that the most frequent issues with hospitalized HIV-infected patients are caused by combinations of contraindicated or not recommended drugs and by dosage errors, which happen in approximately one in five patients [5].

During one year of study, compared to the control group, the intervention group showed higher percentage of clinical outcomes, however there was no statistical difference. The better clinical response is associated with slower disease progression and a lower risk of complications, opportunistic infections, and co-infections [1,30-32]. We speculate that owing to these better clinical outcomes, the intervention group needed less hospitalization, laboratory tests, and medical appointments than the control group did.

Even though the difference in costs between the groups was not statistically significant, we can expect to see an overall, long-term cost analysis for the intervention group due its better clinical outcomes than the control group.

The lower costs associated with appointments and hospitalizations generated by the intervention group, compared with those of the control group, are consistent with the literature. Horberg et al. [13] and McPherson-Baker et al. [33] showed that the pharmacist’s presence may decrease the number of appointments and, therefore, the costs for HIV-infected patients. A systematic review that included 32 articles pertaining to the impact of pharmaceutical care on HIV-infected patients showed that pharmaceutical care is associated with cost savings because it decreases the number of physician appointments, hospitalizations, and emergency visits [9]. A study in China found that total hospitalization costs in a group undergoing pharmaceutical care were significantly lower than those in a control group ($1,442.3 [684.9] vs. $1,729.6 [773.7], P <0.001) [34]. Furthermore, a Taiwanese study showed that the replacement of intravenous levofloxacin with its oral form, performed by a pharmacist, decreased hospital stays from 27.2 to 16.1 days (P =0.001), thereby lowering hospital costs [35]. Nevertheless, we found no studies in the literature that described the impact that pharmaceutical care has on the costs associated with laboratory tests and procedures.

In this study, an economic analysis that correlates the effectiveness and the costs of pharmacotherapy demonstrated that pharmaceutical care is dominant (less expensive and more effective), when we consider the effectiveness outcomes and the costs associated with appointments, laboratory tests, and hospitalizations. However, the intervention group generated higher costs associated with procedures and total costs than those of the control group. Furthermore, this study demonstrates the importance of considering the costs associated with procedures. Here, if costs of procedures were disregarded when calculating the daily costs, the total costs of the intervention group would have been lower than those of the control group.

Cost analysis identified a negative relationship when considering total cost, which contradicts the literature. According to Brennan et al., a $1.00 investment in pharmaceutical care showed a $3.00 return [36]. A meta-analysis demonstrated that 85% of the studies describe positive economic impacts of pharmaceutical care and concluded that the median benefit:cost ratio was 4.68:1 [10]. However, it should be noted that costs associated with laboratory tests and procedures were not included in these studies. In our study, by disregarding the procedures cost, the relationship becomes positive (2.51:1). Inclusion of costs associated with laboratory tests and procedures explains why this study showed different results than the results from the literature, and these differences clarify the need for well-designed studies that include the costs associated with procedures and laboratory tests, for a better understanding of the relationships among pharmaceutical care, HIV-infected patients, and the economy.

### Limitations

This study has some limitations. There was no randomization of patients; the pharmacy staff was not blinded; pharmacotherapy problems were not verified for the control group (it was considered unethical to identify pharmacotherapy problems without providing any intervention); and the biggest limitation was our inability to retrieve the costs associated with the use of drugs, due to lack of information in the patient’s medical charts. Several studies have analyzed the costs associated with the use of drugs [9,11-13,37] because they make a significant contribution to health care costs, especially in the context of hospital care, which represents 15%–25% of total health care costs. In a study conducted at the Maine Medical Center to guide the use of antibiotics, the pharmacist performed 74 interventions, which reduced the costs associated with antibiotics use, especially by replacing parenteral with oral formulations, generating savings of approximately $400.00 per patient and decreasing the length of hospital stay [38]. Therefore, with more comprehensive patient data, important additional savings regarding the use of drugs to treat co-infections could have been verified, since the intervention group had fewer co-infections than the control group did. For example, tuberculosis is common co-infection among HIV-infected patients and its treatment consists of a combination of rifampicin, isoniazid, and pyrazinamide [32], generating a cost of $316.56 per patient [39].

## Conclusion

Our study presents important information about the impact that pharmaceutical care of HIV-infected patients can have on costs associated with procedure and laboratory tests. This information could not be found elsewhere in the literature, which indicates the need for well-designed and more complete studies.

This work demonstrated that pharmaceutical care of HIV-infected patients, for a one-year period, was able to decrease the number of pharmacotherapy problems. In addition, the intervention group presented higher percentage of better clinical outcomes and lower costs associated with appointments, laboratory tests, and hospitalizations than control group, however, there was no statistical difference; and, conversely, higher total costs and costs associated with procedures than those of the control group (no statistical significance). Additional pharmacoeconomic studies focused on pharmaceutical care are necessary to achieve a more comprehensive and reliable analysis.
